# Supplementary material for: A clinical predictive model for pre-transplantation Klebsiella pneumoniae colonization and relevance for clinical outcomes in patients receiving allogeneic hematopoietic stem cell transplantation
Source: Microbiol Spectr. 2024 Jan 8;12(2):e02039-23. doi: 10.1128/spectrum.02039-23 (PMC10846164; doi:10.1128/spectrum.02039-23)

Supplementary Materials

Table S1.Clinical characteristics of patients in derivation cohort (n=448) and validation cohort (n=299).

| Characteristics | Derivation cohort (n=448) | Validation cohort (n=299) | P value |
| --- | --- | --- | --- |
| **Gender, no. of males (%)** | 246(54.9) | 185(61.9) | 0.070 |
| **Age, years, median（IQR）** | 36(27,48) | 37(27,47) | 0.667 |
| **HCT-CI** |  |  | 0.975 |
| ≥3 | 21(4.7) | 15(5.0) |  |
| ＜3 | 426(95.3) | 284(95.0) |  |
| **Underlying disease, n (%)** |  |  | 0.928 |
| AML | 204(45.5) | 132(44.1) |  |
| ALL | 116(25.9) | 84(28.1) |  |
| MDS | 55(12.3) | 32(10.7) |  |
| AA | 19(4.2) | 14(4.7) |  |
| Others | 54(12.1) | 37(12.4) |  |
| **Days from Diagnosis to HSCT,**  **Median (IQR)** | 178(128,246) | 185(135,245) | 0.541 |
| **Times of chemotherapy**  **or hospitalization，Median (IQR)** | 3(2,4) | 3(2,4) | 0.551 |
| **CarT therapy pre-allo-HSCT** | 40(8.9) | 28(9.4) | 0.942 |
| **HLA antigen positive** | 48(10.7) | 28(9.4) | 0.635 |
| **Plasma exchange** | 26(5.8) | 13(4.3) | 0.479 |
| **CD20 therapy** | 47(10.5) | 28(9.4) | 0.706 |
| **Disease status at HSCT** |  |  | 0.979 |
| CR | 373(83.3) | 150(83.6) |  |
| Not CR | 75(16.7) | 49(16.4) |  |
| **Donor** |  |  | 0.100 |
| Haploidentical | 287(64.1) | 210(70.2) |  |
| MSD | 88(19.6) | 56(18.7) |  |
| MUD | 73(16.3) | 33(11.0) |  |
| **Conditioning regimen** |  |  | 0.980 |
| Myeloablative | 418(93.3) | 278(93.0) |  |
| RIC or NMA | 30(6.7) | 21(7.0) |  |
| **Graft source** |  |  | 0.113 |
| PB only | 276(61.6) | 166(55.5) |  |
| BM | 172(38.4) | 133(44.5) |  |
| **ABO incompatibility** |  |  | 0.446 |
| Compatible | 253(56.5) | 166(55.5) |  |
| Minor mismatch | 119(26.6) | 72(24.1) |  |
| Major/bidirectional mismatch | 76(17.0) | 61(20.4) |  |
| **Donor-recipient gender match** |  |  | 0.085 |
| Female to male | 63(14.1) | 242(80.9) |  |
| Others | 385(85.9) | 57(19.1) |  |
| **MNC (10E8/kg), median (IQR)** | 9.11(6.71,12.08) | 9.14(6.68,12.38) | 0.745 |
| **CD34+(10E6/kg),** **median** **(IQR)** | 3.90(2.98,5.35) | 3.93(3.00,5.12) | 0.793 |

HCT-CI, hematopoietic cell transplantation–comorbidity index; AML, acute myelogenous leukemia; ALL, acute lymphoblastic leukemia; MDS, myelodysplastic; AA, aplastic anemia; CAR-T, chimeric antigen receptor-engineered (CAR)-T cell immunotherapy; MSD, matched sibling donor; MUD, matched unrelated donor; CR, complete remission; MAC, myeloablative conditioning; RIC, reduced intensity conditioning; NMA, non-myeloablative conditioning; BM, bone marrow; PB, peripheral blood; MNC, mononuclear cells.

Table S2.Analysis of factors affecting 100d relapse in 747 allo-HSCT patients.

| Characteristics | Univariate Analysis | | |  | Multivariate Analysis | | |
| --- | --- | --- | --- | --- | --- | --- | --- |
|  | HR | CI95 | P-value |  | HR | CI95 | P-Value |
| **KP colonization**  No  Yes | 1.50 | 0.70-3.25 | 0.299 |  |  |  |  |
| **Gender** | 0.83 | 0.41-1.66 | 0.592 |  |  |  |  |
| Female |  |  |  |  |  |  |  |
| Male |  |  |  |  |  |  |  |
| **Age** | 1.02 | 1. 00-1.05 | 0.108 |  |  |  |  |
| **Days from Diagnosis to HSCT** | 1.00 | 1.00-1.00 | 0.442 |  |  |  |  |
| **Times of chemotherapy**  **or hospitalization** | 1.10 | 0.99-1.11 | **0.015** |  | 1.06 | 0.97-1.15 | 0.187 |
| **Diagnosis** |  |  |  |  |  |  |  |
| AA |  |  |  |  |  |  |  |
| ALL  AML or MDS | 0.00  1.06 | 0.00-Inf  0.46-2.43 | 0.982  0.897 |  |  |  |  |
| AML | 0.57 | 0.20-1.64 | 0.298 |  |  |  |  |
| MDS | 0.76 | 0.30-1.90 | 0.551 |  |  |  |  |
| Others | 1.29 | 0.45-3.72 | 0.636 |  |  |  |  |
| **Car-T therapy pre-allo-HSCT** | 4.35 | 2.01-9.41 | **<0.001** |  | 3.75 | 1.61-8.77 | **0.002** |
| **HLA antigen positive** | 1.27 | 0.45-3.62 | 0.655 |  |  |  |  |
| **Disease status** | 4.03 | 1.99-8.17 | **<0.001** |  | 4.3` | 2.12-8.76 | **<0.001** |
| CR |  |  |  |  |  |  |  |
| Not CR |  |  |  |  |  |  |  |
| **HCT-CI** | 1.58 | 0.38-6.62 | 0.530 |  |  |  |  |
| ＜3 |  |  |  |  |  |  |  |
| ≥3 |  |  |  |  |  |  |  |
| **Donor** |  |  |  |  |  |  |  |
| Haploidentical |  |  |  |  |  |  |  |
| MSD | 1.29 | 0.57-2.92 | 0.538 |  |  |  |  |
| MUD | 0.65 | 0.19-2.18 | 0.486 |  |  |  |  |
| **ABO incompatibility** |  |  |  |  |  |  |  |
| Compatible |  |  |  |  |  |  |  |
| Minor mismatch | 1.22 | 0.51-2.95 | 0.655 |  |  |  |  |
| Major/bidirectional mismatch | 1.03 | 0.45-2.40 | 0.938 |  |  |  |  |
| **Female to male** | 0.68 | 0.30-1.58 | 0.376 |  |  |  |  |
| No |  |  |  |  |  |  |  |
| Yes |  |  |  |  |  |  |  |
| **Conditioning regimen** | 1.47 | 0.45-4.83 | 0.524 |  |  |  |  |
| MAC |  |  |  |  |  |  |  |
| RIC or NMA |  |  |  |  |  |  |  |
| **Graft souce** |  |  |  |  |  |  |  |
| BM |  |  |  |  |  |  |  |
| PB  PB+BM | 1.13  0.85 | 0.15-8.38  0.11-6.62 | 0.907  0.875 |  |  |  |  |
| **Total MNC (10E8/kg)** | 1.01 | 0.93-1.09 | 0.843 |  |  |  |  |
| **Total CD34+ (10E6/kg)** | 1.05 | 0.91-1.21 | 0.501 |  |  |  |  |

HCT-CI, hematopoietic cell transplantation–comorbidity index; AML, acute myelogenous leukemia; ALL, acute lymphoblastic leukemia; MDS, myelodysplastic; CAR-T, chimeric antigen receptor-engineered (CAR)-T cell immunotherapy; MSD, matched sibling donor; MUD, matched unrelated donor; CR, complete remission; MAC, myeloablative conditioning; RIC, reduced intensity conditioning; NMA, non-myeloablative conditioning; BM, bone marrow; PB, peripheral blood; MNC, mononuclear cells.

Table S3.Analysis of factors affecting 100d NRM in 747 allo-HSCT patients.

| Characteristics | Univariate Analysis | | |  | Multivariate Analysis | | |
| --- | --- | --- | --- | --- | --- | --- | --- |
|  | HR | CI95 | P-value |  | HR | CI95 | P-Value |
| **KP colonization**  No  Yes | 2.44 | 1.32-4.50 | **0.004** |  | 2.39 | 1.26-4.52 | **0.007** |
| **Gender** | 0.92 | 0.51-1.69 | 0.794 |  |  |  |  |
| Female |  |  |  |  |  |  |  |
| Male |  |  |  |  |  |  |  |
| **Age** | 1.04 | 1. 02-1.07 | **0.001** |  | 1.04 | 1.01-1.07 | **0.003** |
| **Days from Diagnosis to HSCT** | 1.00 | 1.00-1.00 | 0.195 |  |  |  |  |
| **Times of chemotherapy**  **or hospitalization** | 1.05 | 0.96-1.14 | 0.297 |  |  |  |  |
| **Diagnosis** |  |  |  |  |  |  |  |
| AA  ALL  AML  MDS | 0.36  0.30  0.54 | 0.11-1.15  0.10-0.92  0.15-1.91 | 0.085  0.035  0.340 |  | 0.41  0.24 | 0.12-1.37  0.07-0.78 | 0.147  0.017 |
| ALL | 0.36 | 0.11-.1.15 | **0.085** |  | 0.41 | 0.12-1.37 | 0.147 |
| AML | 0.30 | 0.10-0.92 | **0.035** |  | 0.24 | 0.07-0.78 | **0.017** |
| MDS | 0.54 | 0.15-1.91 | 0.340 |  |  |  |  |
| Others | 0.99 | 0.31-3.11 | 0.984 |  |  |  |  |
| **Car-T therapy pre-allo-HSCT** | 1.69 | 0.72-4.02 | 0.231 |  |  |  |  |
| **HLA antigen positive** | 0.66 | 0.20-2.13 | 0.485 |  |  |  |  |
| **Disease status** | 3.24 | 1.74-6.01 | **<0.001** |  | 2.61 | 1.38-4.91 | **0.003** |
| CR |  |  |  |  |  |  |  |
| Not CR |  |  |  |  |  |  |  |
| **HCT-CI** | 4.35 | 1.93-9.77 | **<0.001** |  | 2.54 | 1.10-5.85 | **0.029** |
| ＜3 |  |  |  |  |  |  |  |
| ≥3 |  |  |  |  |  |  |  |
| **Donor** |  |  |  |  |  |  |  |
| Haploid |  |  |  |  |  |  |  |
| MSD | 0.64 | 0.27-1.53 | 0.312 |  |  |  |  |
| MUD | 0.73 | 0.28-1.86 | 0.507 |  |  |  |  |
| **ABO incompatibility** |  |  |  |  |  |  |  |
| Compatible |  |  |  |  |  |  |  |
| Minor mismatch | 0.46 | 0.16-1.32 | 0.148 |  |  |  |  |
| Major/bidirectional mismatch | 1.10 | 0.56-2.14 | 0.780 |  |  |  |  |
| **Female to male** | 1.44 | 0.57-3.67 | 0.441 |  |  |  |  |
| Yes |  |  |  |  |  |  |  |
| No |  |  |  |  |  |  |  |
| **Conditioning regimen** | 1.45 | 0.52-4.06 | 0.480 |  |  |  |  |
| Myeloablative |  |  |  |  |  |  |  |
| RIC or NMA |  |  |  |  |  |  |  |
| **Graft souce** |  |  |  |  |  |  |  |
| BM |  |  |  |  |  |  |  |
| PB  PB+BM | 0.61  0.83 | 0.14-2.57  0.19-3.54 | 0.497  0.797 |  |  |  |  |
| **Total MNC (10E8/kg)** | 0.99 | 0.93-1.07 | 0.889 |  |  |  |  |
| **Total CD34+ (10E6/kg)** | 0.98 | 0.85-1.14 | 0.839 |  |  |  |  |

HCT-CI, hematopoietic cell transplantation–comorbidity index; AML, acute myelogenous leukemia; ALL, acute lymphoblastic leukemia; MDS, myelodysplastic; CAR-T, chimeric antigen receptor-engineered (CAR)-T cell immunotherapy; MSD, matched sibling donor; MUD, matched unrelated donor; CR, complete remission; MAC, myeloablative conditioning; RIC, reduced intensity conditioning; NMA, non-myeloablative conditioning; BM, bone marrow; PB, peripheral blood; MNC, mononuclear cells.

Table S4.Analysis of factors affecting 100d PFS in 747 allo-HSCT patients.

| Characteristics | Univariate Analysis | | |  | Multivariate Analysis | | |
| --- | --- | --- | --- | --- | --- | --- | --- |
|  | HR | CI95 | P-value |  | HR | CI95 | P-Value |
| **KP colonization**  No  Yes | 2.27 | 1.48-3.47 | **<0.001** |  | 1.89 | 1.22-2.91 | **0.004** |
| **Gender** | 0.85 | 0.56-1.29 | 0.450 |  |  |  |  |
| Male |  |  |  |  |  |  |  |
| Female |  |  |  |  |  |  |  |
| **Age** | 1.03 | 1. 02-1.05 | **<0.001** |  | 1.03 | 1.02-1.05 | **<0.001** |
| **Days from Diagnosis to HSCT** | 1.00 | 1.00-1.00 | 0.151 |  |  |  |  |
| **Times of chemotherapy**  **or hospitalization** | 1.06 | 1.00-1.12 | **0.033** |  | 1.04 | 0.97-1.11 | 0.249 |
| **Diagnosis** |  |  |  |  |  |  |  |
| AA  ALL  AML  MDS | 0.58  0.56  0.47 | 0.23-1.42  0.23-1.32  0.16-1.36 | 0.230  0.183  0.164 |  |  |  |  |
| ALL | 0.58 | 0.23-1.42 | 0.230 |  |  |  |  |
| AML | 0.56 | 0.23-1.32 | 0.183 |  |  |  |  |
| MDS | 0.47 | 0.16-1.36 | 0.164 |  |  |  |  |
| Others | 1.16 | 0.47-2.92 | 0.744 |  |  |  |  |
| **Car-T therapy pre-allo-HSCT** | 2.30 | 1.34-3.95 | **0.002** |  | 2.39 | 1.31-4.36 | **0.005** |
| **HLA antigen positive** | 0.96 | 0.48-1.92 | 0.916 |  |  |  |  |
| **Disease status** | 3.28 | 2.14-5.03 | **<0.001** |  | 3.00 | 1.95-4.63 | **<0.001** |
| CR |  |  |  |  |  |  |  |
| Not CR |  |  |  |  |  |  |  |
| **HCT-CI** | 2.54 | 1.28-5.06 | **0.008** |  | 1.65 | 0.81-3-34 | 0.165 |
| ＜3 |  |  |  |  |  |  |  |
| ≥3 |  |  |  |  |  |  |  |
| **Donor** |  |  |  |  |  |  |  |
| Haploid |  |  |  |  |  |  |  |
| MSD | 1.01 | 0.60-1.71 | 0.966 |  |  |  |  |
| MUD | 0.90 | 0.49-1.67 | 0.742 |  |  |  |  |
| **ABO incompatibility** |  |  |  |  |  |  |  |
| Compatible |  |  |  |  |  |  |  |
| Minor mismatch | 0.78 | 0.43-1.40 | 0.409 |  |  |  |  |
| Major/bidirectional mismatch | 0.94 | 0.58-1.53 | 0.803 |  |  |  |  |
| **Female to male** | 0.95 | 0.55-1.66 | 0.862 |  |  |  |  |
| Yes |  |  |  |  |  |  |  |
| No |  |  |  |  |  |  |  |
| **Conditioning regimen** | 1.63 | 0.82-3.24 | 0.167 |  |  |  |  |
| Myeloablative |  |  |  |  |  |  |  |
| RIC or NMA |  |  |  |  |  |  |  |
| **Graft souce** |  |  |  |  |  |  |  |
| BM |  |  |  |  |  |  |  |
| PB  PB+BM | 0.96  0.99 | 0.30-3.06  0.31-3.23 | 0.939  0.990 |  |  |  |  |
| **Total MNC (10E8/kg)** | 1.00 | 0.95-1.04 | 0.846 |  |  |  |  |
| **Total CD34+ (10E6/kg)** | 1.02 | 0.93-1.12 | 0.728 |  |  |  |  |

HCT-CI, hematopoietic cell transplantation–comorbidity index; AML, acute myelogenous leukemia; ALL, acute lymphoblastic leukemia; MDS, myelodysplastic; CAR-T, chimeric antigen receptor-engineered (CAR)-T cell immunotherapy; MSD, matched sibling donor; MUD, matched unrelated donor; CR, complete remission; MAC, myeloablative conditioning; RIC, reduced intensity conditioning; NMA, non-myeloablative conditioning; BM, bone marrow; PB, peripheral blood; MNC, mononuclear cells.

Table S5.Analysis of factors affecting 100d OS in 747 allo-HSCT patients.

| Characteristics | Univariate Analysis | | |  | Multivariate Analysis | | |
| --- | --- | --- | --- | --- | --- | --- | --- |
|  | HR | CI95 | P-value |  | HR | CI95 | P-Value |
| **KP colonization**  Yes  No | 2.09 | 1.15-3.78 | **0.015** |  | 1.93 | 1.04-3.60 | **0.038** |
| **Gender** | 0.91 | 0.51-1.61 | 0.741 |  |  |  |  |
| Male |  |  |  |  |  |  |  |
| Female |  |  |  |  |  |  |  |
| **Age** | 1.04 | 1. 01-1.06 | **0.002** |  | 1.04 | 1.02-1.07 | **0.001** |
| **Days from Diagnosis to HSCT** | 1.00 | 1.00-1.00 | **0.047** |  | 1.00 | 1.00-1.00 | 0.717 |
| **Times of chemotherapy**  **or hospitalization** | 1.05 | 0.97-1.14 | 0.244 |  |  |  |  |
| **Diagnosis** |  |  |  |  |  |  |  |
| AA  ALL  AML  MDS | 0.48  0.33  0.55 | 0.16-1.50  0.11-1.01  0.16-1.96 | 0.209  0.052  0.361 |  | 0.29 | 0.09-0.93 | 0.037 |
| ALL | 0.48 | 0.16-1.50 | 0.209 |  |  |  |  |
| AML | 0.33 | 0.11-1.01 | **0.052** |  | 0.29 | 0.09-0.93 | **0.037** |
| MDS | 0.55 | 0.16-1.96 | 0.361 |  |  |  |  |
| Others | 1.01 | 0.32-3.18 | 0.983 |  |  |  |  |
| **Car-T therapy pre-allo-HSCT** | 2.14 | 1.00-4.59 | **0.049** |  |  |  |  |
| **HLA antigen positive** | 0.59 | 0.18-1.91 | 0.382 |  |  |  |  |
| **Disease status** | 4.03 | 2.26-7.18 | **<0.001** |  | 3.16 | 1.72-5.80 | **<0.001** |
| CR |  |  |  |  |  |  |  |
| Not CR |  |  |  |  |  |  |  |
| **HCT-CI** | 3.97 | 1.78-8.87 | **0.001** |  | 2.22 | 0.96-5.13 | 0.063 |
| ＜3 |  |  |  |  |  |  |  |
| ≥3 |  |  |  |  |  |  |  |
| **Donor** |  |  |  |  |  |  |  |
| Haploid |  |  |  |  |  |  |  |
| MSD | 0.93 | 0.44-1.93 | 0.837 |  |  |  |  |
| MUD | 0.71 | 0.28-1.81 | 0.466 |  |  |  |  |
| **ABO incompatibility** |  |  |  |  |  |  |  |
| Compatible |  |  |  |  |  |  |  |
| Minor mismatch | 0.41 | 0.14-1.17 | **0.097** |  | 0.45 | 0.16-1.30 | 0.141 |
| Major/bidirectional mismatch | 1.06 | 0.56-2.01 | 0.849 |  |  |  |  |
| **Female to male** | 1.61 | 0.64-4.07 | 0.314 |  |  |  |  |
| Yes |  |  |  |  |  |  |  |
| No |  |  |  |  |  |  |  |
| **Conditioning regimen** | 1.65 | 0.65-4.18 | 0.287 |  |  |  |  |
| Myeloablative |  |  |  |  |  |  |  |
| RIC or NMA |  |  |  |  |  |  |  |
| **Graft souce** |  |  |  |  |  |  |  |
| BM |  |  |  |  |  |  |  |
| PB  PB+BM | 0.68  0.86 | 0.16-2.87  0.20-3.68 | 0.601  0.838 |  |  |  |  |
| **Total MNC (10E8/kg)** | 0.99 | 0.93-1.06 | 0.880 |  |  |  |  |
| **Total CD34+ (10E6/kg)** | 1.03 | 0.91-1.17 | 0.624 |  |  |  |  |

HCT-CI, hematopoietic cell transplantation–comorbidity index; AML, acute myelogenous leukemia; ALL, acute lymphoblastic leukemia; MDS, myelodysplastic; CAR-T, chimeric antigen receptor-engineered (CAR)-T cell immunotherapy; MSD, matched sibling donor; MUD, matched unrelated donor; CR, complete remission; MAC, myeloablative conditioning; RIC, reduced intensity conditioning; NMA, non-myeloablative conditioning; BM, bone marrow; PB, peripheral blood; MNC, mononuclear cells.

Table S6.Causes of death of KP-colonization group and Non-colonization group.

| Characteristics | KP-colonization group (44/166,26.5%) | Non-colonization group (98/581,16.8%) |
| --- | --- | --- |
| Relapse | 14 | 30 |
| Secondary infection | 23 | 27 |
| aGVHD | 10 | 12 |
| TMA | 3 | 9 |
| VOD | 1 | 1 |
| CNS disease | 6 | 14 |
| DAH | 0 | 1 |
| Liver failure | 0 | 2 |
| Cardiopulmonary arrest | 0 | 1 |
| Acute pancreatitis | 0 | 1 |
| Gastrointestinal bleeding | 2 | 7 |
| Cardiac events | 0 | 2 |

When the patient dies of two unidentifiable causes, both are recorded as 1.

aGVHD, acute graft-versus-host disease; TMA, thrombotic microangiopathy; VOD, hepatic vein occlusive disease; CNS, central nervous system; DAH, diffuse alveolar hemorrhage.

Table S7.Causes of death within 100 days after allo-HSCT of KP-colonization group and Non-colonization group.

| Characteristics | KP-colonization group (16/166,9.6%) | Non-colonization group (31/581,5.3%) |
| --- | --- | --- |
| Relapse | 0 | 7 |
| Secondary infection | 9 | 6 |
| aGVHD | 5 | 7 |
| TMA | 3 | 5 |
| VOD | 1 | 0 |
| CNS disease | 4 | 6 |
| DAH | 0 | 1 |
| Cardiopulmonary arrest | 0 | 1 |
| Gastrointestinal bleeding | 2 | 3 |
| Cardiac events | 0 | 1 |

When the patient dies of two unidentifiable causes, both are recorded as 1.

aGVHD, acute graft-versus-host disease; TMA, thrombotic microangiopathy; VOD, hepatic vein occlusive disease; CNS, central nervous system; DAH, diffuse alveolar hemorrhage.


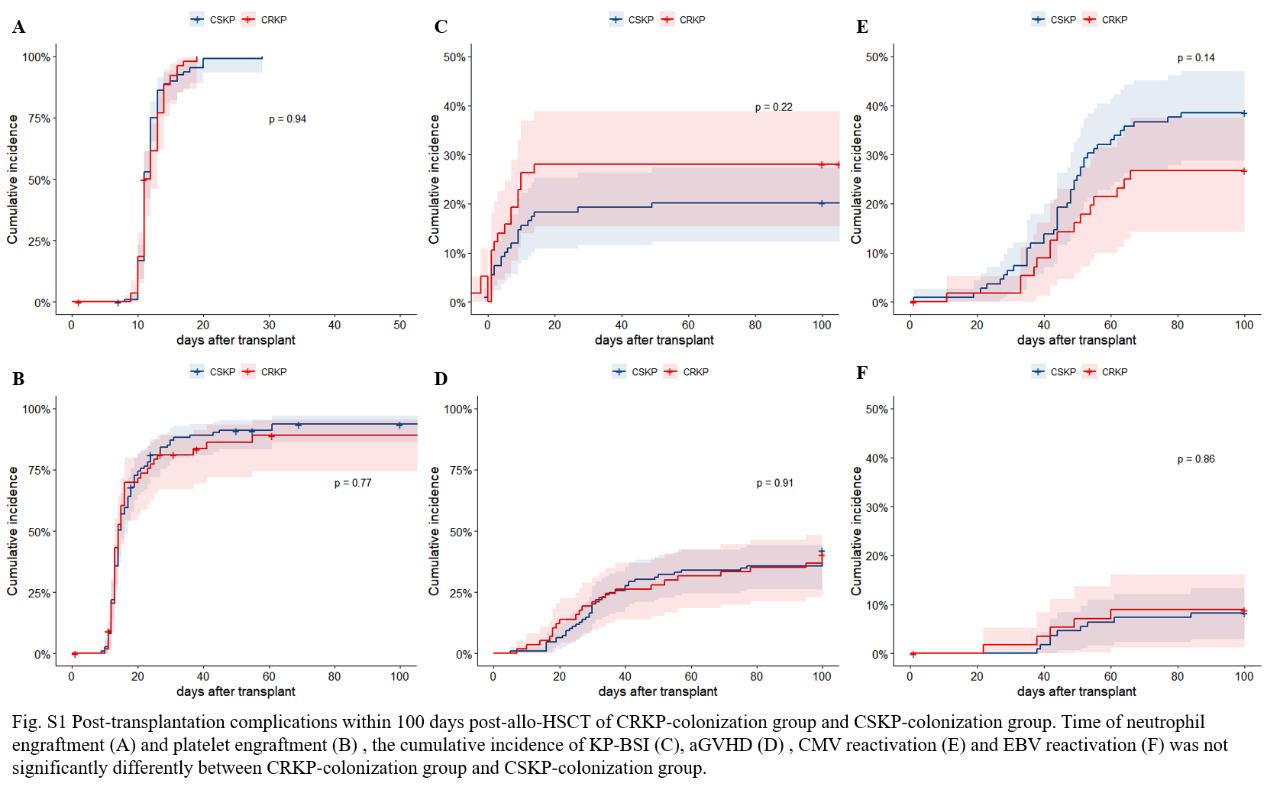

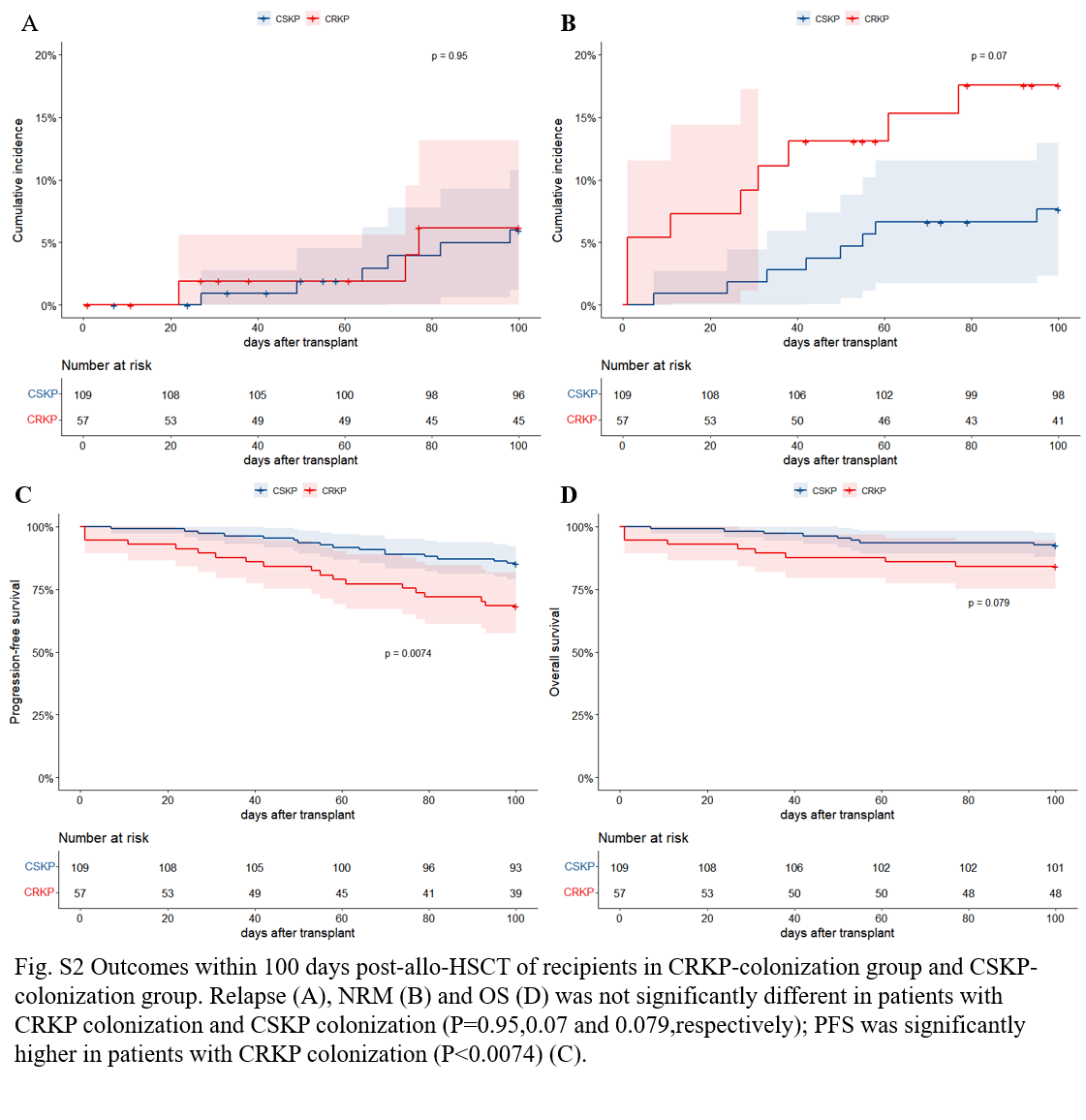

Supplement: Supplemental material — Tables S1 to S7; Fig. S1 and S2. [file spectrum.02039-23-s0001.docx]
